# Listeria monocytogenes meningitis in immunocompetent and healthy children: a case report and a review of the literature

**DOI:** 10.1186/s13052-018-0595-5

**Published:** 2018-12-29

**Authors:** Massimo Luca Castellazzi, Paola Marchisio, Samantha Bosis

**Affiliations:** Pediatric Highly Intensive Care Unit, Department of Pathophysiology and Transplantation, Università degli Studi di Milano, Fondazione IRCCS Ca’ Granda Ospedale Maggiore Policlinico, Milan, Italy

**Keywords:** *Listeria monocytogenes*, Meningitis, Immunocompetent child

## Abstract

**Background:**

*Listeria monocytogenes* is a gram-positive bacteria generally transmitted to humans through ingestion of contaminated food. It typically infects high risk subjects, such as pregnant women, neonates, the elderly and immunocompromised patients. Listeria meningitis is rarely reported in previously healthy children with no immunological disorders. However, it can be aggressive in such subjects and is associated with a high mortality rate. Prompt diagnosis is essential so that adequate antibiotic treatment can be started and the best outcome achieved.

**Case presentation:**

We report the case of a previously healthy 16-month-old child with Listeria meningitis who was successfully treated with intravenous ampicillin and gentamicin without any sequelae.

**Conclusions:**

Although Listeria meningitis is rare in previously healthy immunocompetent children, it must be considered, especially in children who do not improve with first-line antibiotic treatment. A review of the literature published since 1996 has been performed, to provide a general overview on this topic.

## Background

*Listeria (L.) monocytogenes* is a gram-positive facultative intracellular bacteria transmitted to humans through ingestion of contaminated food, in particular ready-to-eat food, products with a long shelf life, deli meats and soft cheeses. It typically affects pregnant women, neonates, the elderly and immunocompromised patients [1]. An infection during pregnancy can cause abortion, premature birth and amniositis, whereas in neonates it can cause late-onset meningitis, conjunctivitis and pneumonia. In immunocompromised patients Listeria causes central nervous system infection, endocarditis, and sepsis [2]. There are rare reports of Listeria meningitis in previously healthy and immunocompetent children, which may be associated with severe complications and a high mortality rate [2].

The clinical symptoms of Listeria meningitis are similar to those of other causes of meningoencephalitis and first-line treatment with third-generation cephalosporins is ineffective. Awareness of this pathogen is therefore crucial, to enable adequate treatment to be started and the best outcome to be achieved.

We report on a case of Listeria meningitis in a previously healthy 16-month-old girl. We also review literature reports of this condition in post-neonatal immunocompetent children, published in English since 1996, in order to discuss its clinical presentation, diagnosis, treatment options and prognosis.

## Case presentation

A previously healthy, fully immunized, 16-month-old girl was hospitalized for high grade fever (maximum axillary temperature of 39.8 °C), vomiting and refusal to feed of 4 days’ duration and irritability of recent onset. She had been receiving oral antibiotic treatment with amoxicillin-clavulanic acid (50 mg/kg/day every 8 h) for 24 h, without any clinical improvement. On admission (day 1) the patient was irritable but in good general condition. Physical findings were as follows: body weight 11 kg; heart rate 101 beats/min; body temperature 38 °C; oxygen saturation in room air 98%; blood pressure 90/50 mmHg. The cardiorespiratory and abdominal examinations were normal and no skin rash was observed. There was no sign of meningitis. Laboratory tests showed elevated white blood cell (WBC) counts of 14,090/mmc (75.7% neutrophils), while the C-reactive protein (CRP) concentration was 5.76 mg/dl (normal value < 0.5 mg/dl). Electrolytes, renal function and coagulation tests were within the normal range. Given her vomiting, the ongoing oral antibiotic treatment was stopped and intravenous ceftriaxone (100 mg/kg/day in a single dose) was started in the suspicion of a bacterial infection.

On day 2 the patient rapidly worsened. She was lethargic and preferred the lying position. Neck stiffness and Brudzinski’s sign were also noted. A computed tomography scan of the brain was normal, with no signs of increased intracranial pressure. A lumbar puncture was performed, revealing clear cerebrospinal fluid (CSF) containing 840 cells/mmc with neutrophilic predominance and glucose and protein concentrations of 38 and 44 mg/dl respectively. Empiric parenteral antibiotic treatment with ceftriaxone (100 mg/kg/day) was continued and intravenous antiviral therapy with acyclovir (30 mg/kg/day in 3 doses) was started. Gram-staining resulted negative.

On day 3, a real-time polymerase chain reaction (RT-PCR) for viruses and bacteria was positive for *L. monocytogenes*. Ceftriaxone was therefore discontinued and intravenous ampicillin (200 mg/kg/day in 4 doses) and gentamicin (5 mg/kg/day) were started.

On day 4, the CSF culture identified *L. monocytogenes*, while the blood culture was negative. A brain magnetic resonance imaging scan showed mild meningeal enhancement without any sign of parenchymal involvement.

The patient improved rapidly after the initiation of ampicillin and gentamicin. She was completely afebrile from day 7 and was progressively alert and communicative by day 10. Ampicillin and gentamicin were continued for a total of 21 days, while acyclovir was continued until the RT-PCR for Herpes simplex viruses proved negative. The patient was discharged after 22 days in good general condition and without any neurologic sequelae. Immunological screening, including an evaluation of cellular immunity (total and subpopulations of T cells), humoral immunity (immunoglobulin levels and subclass IgG) and complement (C3, C4, AP50, CH50), was normal. An HIV test was negative and there was no iron overload. A hearing test (auditory evoked potentials) was normal. At a follow-up visit after 1 month, her clinical presentation was normal and there were no signs of the disease. No source of infection was clarified in her recent history.

## Discussion and conclusion

*L. monocytogenes* meningoencephalitis in previously healthy immunocompetent children is rare, but it can progress rapidly and may be associated with severe complications, such as acute hydrocephalus, and a high mortality rate [3]. Early diagnosis and adequate treatment are therefore essential to achieve the best outcome. Unfortunately, adequate therapy might be delayed, and it is essential that Listeria meningitis is considered in cases that do not improve following first-line treatment with extended-spectrum cephalosporins for central nervous system infections.

To discuss the clinical presentation, diagnosis, treatment and prognosis of this disease, we performed a literature review of Listeria meningitis in previously healthy children without any immunological disorders, in order to help pediatricians promptly recognize and treat it. We identified 16 case reports involving 21 patients with Listeria meningitis published in English since 1996. Most of these cases shows similarities with our own case report.

The clinical presentation of Listeria meningitis is non-specific and similar to that seen with other types of viral or bacterial meningitis. The signs and symptoms include fever, headache, vomiting, diarrhea and altered mental status [1–16], as also seen in our case. Abducens nerve palsy and nystagmus have also been reported among the initial signs of clinical presentation in some cases [8, 10, 12, 13]. Listeria meningitis was preceded by rotavirus gastroenteritis and by meningococcal B meningitis in two cases [13, 14]. The age at presentation varied from 7 months to 10 years. Most children, including our patient, presented with leukocytosis with neutrophilia and with elevated CRP. Lumbar puncture is essential for diagnosis, as blood cultures are often negative: only 5 cases had a blood culture positive for *L. monocytogenes* [2, 3, 5, 8, 12]. CSF analysis reveals in most cases pleocytosis with neutrophilia, reduced glucose concentration and increased protein levels.

Gram-staining is of limited use as it is negative in most cases. However, positivity for bacillus bacteria should not be automatically considered as a contaminant. In our case, gram-staining was negative, but RT-PCR was rapidly positive for *L. monocytogenes*, confirming the utility of this technique in identifying this pathogen and in guiding adequate antibiotic therapy [17, 18]. Initial CSF gram-staining and culture were reported as negative in a few cases at the first lumbar puncture [2, 3, 6]. Repetition of the CSF analysis is therefore advised in cases of severe meningoencephalitis that do not improve with first-line antibiotic treatment where no specific organism was identified on initial evaluation [2].

Finally, in one case diagnosis was based on serologic tests [9]. However, serologic testing for Listeria meningitis lacks specificity and should only be used for retrospective diagnosis [15].

First-line empiric treatment of meningitis frequently includes third-generation cephalosporins and vancomycin to target the most common pathogens [19]. However, once *L. monocytogenes* has been isolated from the CSF, the treatment should be adjusted to include ampicillin alone or in combination with an aminoglycoside, such as gentamicin or amikacin. Only one in-vitro study has demonstrated that this association is synergic [20]. Although vancomycin may be effective against *L. monocytogenes* on in-vitro testing, it has had a high failure rate clinically [2]. Furthermore, its penetration in CSF is poor [21].

Trimethoprim-sulfamethoxazole was recently described as an effective alternative in CNS listeriosis refractory to conventional treatment [12]. Carbapenems, used alone or in combination with aminoglycoside, have also produced good results [3, 5, 6]. The treatment duration varied from 10 days to a maximum of 8 weeks, depending on the severity of the case. In our case, 21 days of ampicillin and gentamicin were sufficient and the patient was discharged without any sequelae. Table 1 summarizes the main characteristics of Listeria meningitis in healthy and immunocompetent children.

**Table 1:** Listeria meningitis in healthy and immunocompetent children

Author, year of publication, nation, reference	Patient, age (y/m), sex (M/F)	Specific antibiotic treatment for *Listeria*	Duration of antibiotic treatment for *Listeria*	Complications	Outcome
Schneeberger et al., 1996, The Netherlands [14]	30 m, F	Amoxicillin tobramycin	14 days 4 days	None	Discharge
Economou et al., 2000, Greece, [4]	14 m, M 3 y, F	Ampicillin-gentamicin NA	4 weeks NA	None Seizures	Discharge Death
Hitomi et al., 2004, Japan, [5]	2 y, M	Ampicillin-gentamicin, PB, TMP	20 days	None	Discharge
Ulloa-Gutierrez at al., 2004, Costa Rica, [3]	10 y, F 3 y, M 6 y, M	Meropenem, amikacin, rifampin Ampicillin-gentamicin Ampicillin-gentamicin	22, 10, 14 days NA NA	Seizures; Hydrocephalus Hydrocephalus Hydrocephalus	Discharge Death Death
Dilber et al., 2009, Turkey, [6]	7 y, M 18 m, M	Ampicillin-amikacin Meropenem, vancomycin, ampicillin	21–14 days 21, 21, 14 days	None SIADH; abducens palsy	Discharge Discharge
Lee et al., 2010, Korea, [2]	7 y, F	Ampicillin was added to the previous treatment	NA	Hydrocephalus	Discharge
Peer et al., 2010, India, [7]	20 m, F	Ampicillin, amikacin	21 days	Seizures; bilateral rectus palsy	Discharge: 5 months later partial left lateral rectus palsy
Ben Shimol et al., 2011, Israel, [8]	6 y, F	Ampicillin, gentamicin	4 weeks ampicillin 12 days gentamicin	Hydrocephalus	Discharge
Lobotkovà et al., 2014, Slovakia, [9]	2 y, M	Ampicillin	6 weeks	None	Discharge
Gee et al., 2015, USA, [10]	2 y, M	NA	NA	Increased ICP, bradyarrhythmia and hypotension	Discharge (mild speech delay)
Papandreu et al., 2016, UK, [11]	3 y, F	Ampicillin Amikacin Ampicillin Vancomycin Ceftriaxone	27 days 20 days 3 weeks	AIDP	Discharge
Polat et al., 2016, Turkey, [12]	7 m, F	Ampicillin, gentamicin, subsequently replaced with TMP	26 days ampicillin 21 days TMP	None	Discharge
Ohnishi et al., 2017, Japan, [13]	20 m, F	Ampicillin Gentamicin	26 days 21 days	None	Discharge
Villa et al., 2017, Italy, [1]	7 y, M	Ampicillin Gentamicin	20 days 15 days	Left abducens nerve palsy	Discharge: persistence of left abducens palsy 3 weeks later
Shimbo et al., 2018, Japan, [15]	10 y, F 6 y, M	Ampicillin Ampicillin	Not reported Not reported	MERS TCM, left abducens nerve palsy	Discharge Discharge
Xu et al., 2018, China, [16]	2 y, M	Ampicillin Vancomycin	Not reported	MERS	Discharge

*WBC* White blood cells, *CRP* C-reactive protein, *NA* not available, *PB* panipenem-betamipron, *TMP* trimethoprim-sulfametoxazole, *SIADH* secretion of inappropriate antidiuretic hormone, *AIDP* acute inflammatory demyelinating polyneuropathy, *MERS* mild encephalitis/encephalopathy with a reversible splenial lesion, *TCM* takotsubo cardiomyopathy

As *L. monocytogenes* mainly affects immunocompromised hosts, an immunological evaluation could be useful. Underlying causes of immunosuppression, such as prolonged or inappropriate corticosteroid treatment, should be excluded [9]. Finally, blood iron levels should be checked, as iron overload could predispose to Listeria meningitis [22].

*L. monocytogenes* is a rare cause of meningoencephalitis in previously healthy, immunocompetent children. However, its clinical presentation is similar to that of other viral or bacterial CNS infections and its course can be rapid and aggressive. Physicians should therefore always consider *L. monocytogenes* as a possible etiologic agent of meningoencephalitis, especially in cases that are unresponsive to the empiric first-line antibiotic treatment and when gram-positive bacilli are observed in the CSF. Furthermore, real-time PCR, as in our case, may be useful for prompt diagnosis, which is essential to enable adequate antibiotic treatment to be started with ampicillin alone or in combination with aminoglycoside.

## Abbreviations

CRP: C-reactive protein
CSF: Cerebrospinal fluid
L: Listeria
RT-PCR: Real-time polymerase chain reaction
WBC: White blood cell count
